# Supplementary material for: Engineered and hybrid human megakaryocytic extracellular vesicles for targeted non-viral cargo delivery to hematopoietic (blood) stem and progenitor cells
Source: Front Bioeng Biotechnol. 2024 Sep 25;12:1435228. doi: 10.3389/fbioe.2024.1435228 (PMC11461334; doi:10.3389/fbioe.2024.1435228)
Supplement: Supplementary file 1 [file DataSheet1.docx]

Supplementary Material for

**Engineered and hybrid human megakaryocytic extracellular vesicles for targeted non-viral cargo delivery to hematopoietic (blood) stem and progenitor cells**

**Samik Das^1,2^, Will Thompson^1,2^, E. Terry Papoutsakis^1,2,3^***

^1^Department of Chemical and Biomolecular Engineering, University of Delaware, Newark, DE, USA

^2^Delaware Biotechnology Institute, University of Delaware, Newark, DE, USA

^3^Department of Biological Sciences, University of Delaware, Newark, DE, USA

*** Correspondence:**Eleftherios Terry Papoutsakis

Ammon-Pinizzotto Biopharmaceutical Innovation Building

590 Avenue 1743, Newark, DE 19713, USA

epaps@udel.edu

# Supplementary Data

**Table S1:** Mice counts per condition.

**Figure S2:** Development of mature megakaryocytes in murine bone marrow following huMkEV treatment.

**Figure S3:** Impact of liposome, CHEV-liposome hybrid treatment on platelet counts

**Figure S4:** Flushed marrow (femur) cells from mice treated with pDNA –loaded CHEV-liposome hybrids exhibit expression of miRFP703 from delivered plasmid.

**Figure S5:** Histological analysis of various Sca-1 immunostained tissues excised from pDNA-loaded liposome and CHEV-liposome hybrid-treated mice.

**Figure S6:** Histological analysis of various CD41 immunostained tissues excised from pDNA-loaded liposome and CHEV-liposome hybrid-treated mice.

# Supplementary Figures and Tables

## Supplementary Table S1


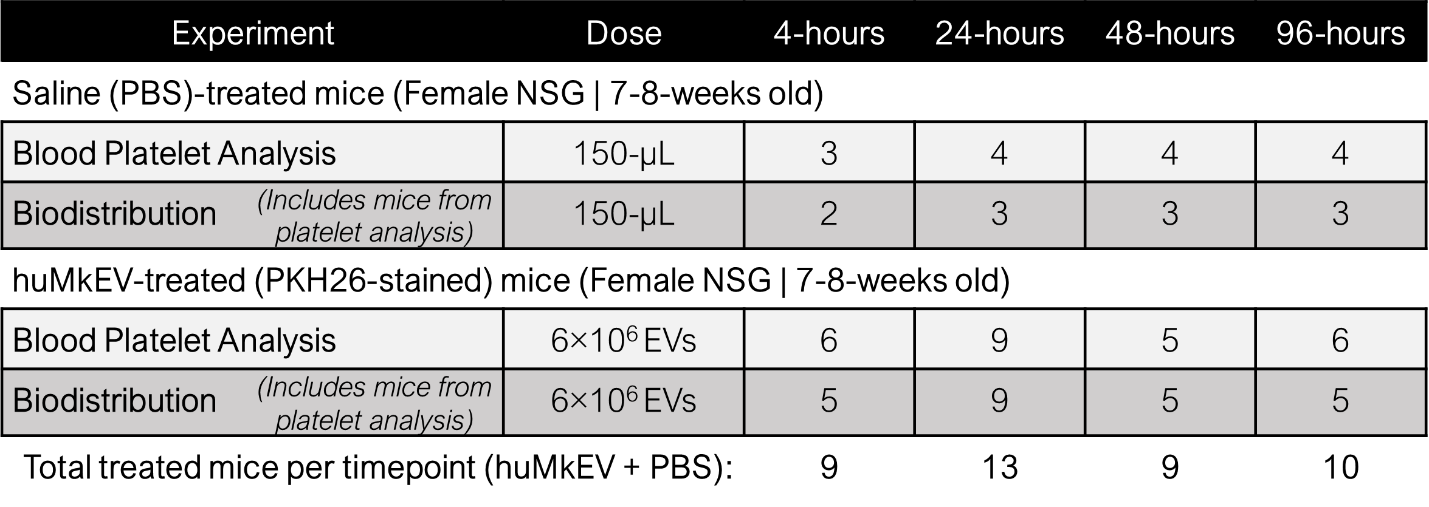


**Supplemental Table S1. Mice counts per condition.** Count of 7-8-week female NSG mice for assessing *in vivo* huMkEV-induced megakaryopoiesis and EV biodistribution at 4-, 24-, 48-, and 96-hours following intravenous administration.

## Supplementary Figure S2
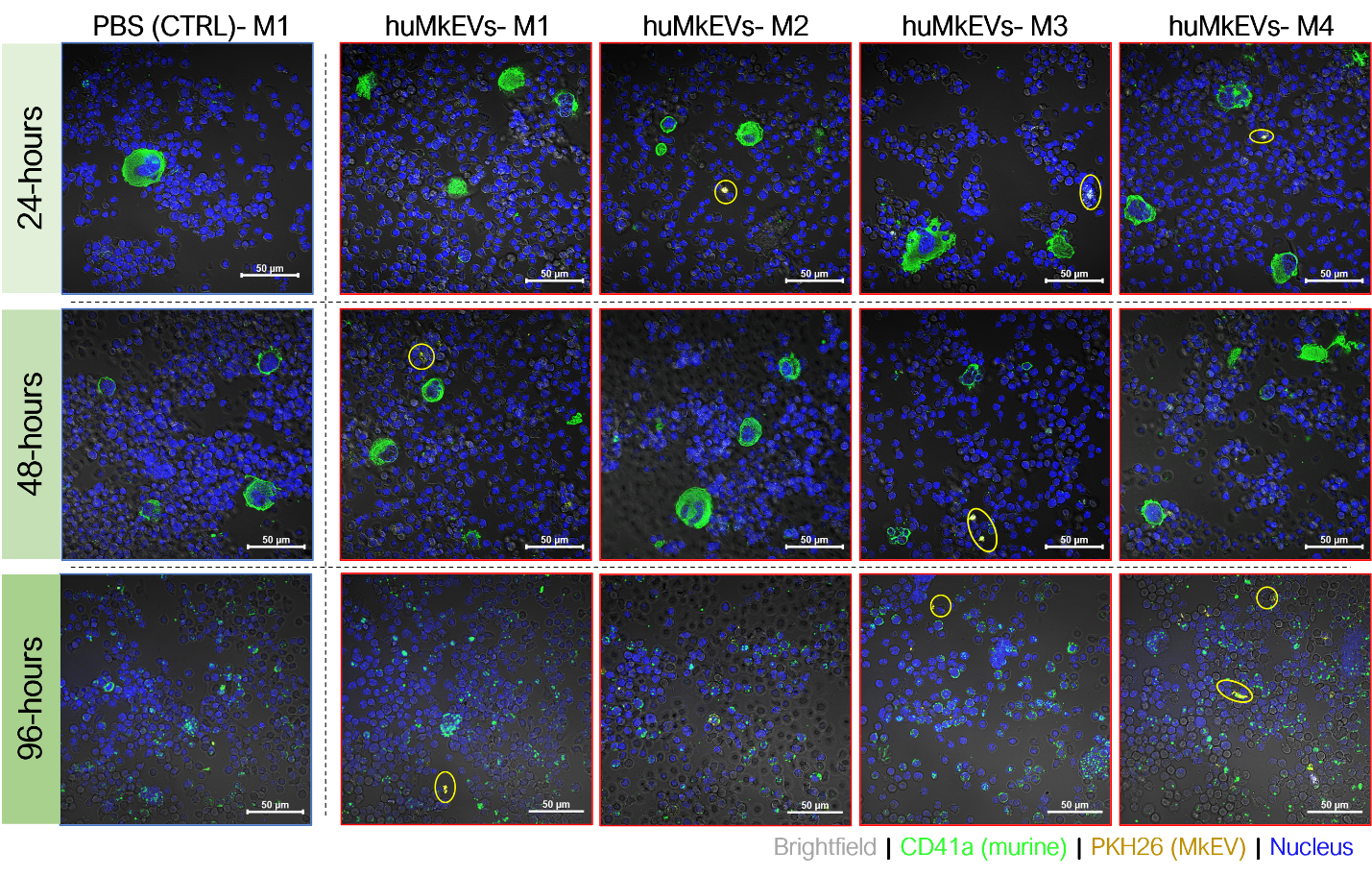


**Supplemental Figure S2.** **Development of mature megakaryocytes in murine bone marrow following huMkEV treatment.** Select bone marrow flushes from PBS-treated (left column) and huMkEV-treated (right 4 columns) mice from 24-hours (top row), 48-hours (middle row), and 96-hours (bottom row) were immunostained for murine CD41a (green) to determine the degree of differentiation of the murine HSPCs to the megakaryocytic phenotype. Presence of huMkMPs indicated in yellow (PKH26) and nuclei are shown in blue (DAPI). Scale bars: 50-µm.

## Supplementary Figure S3


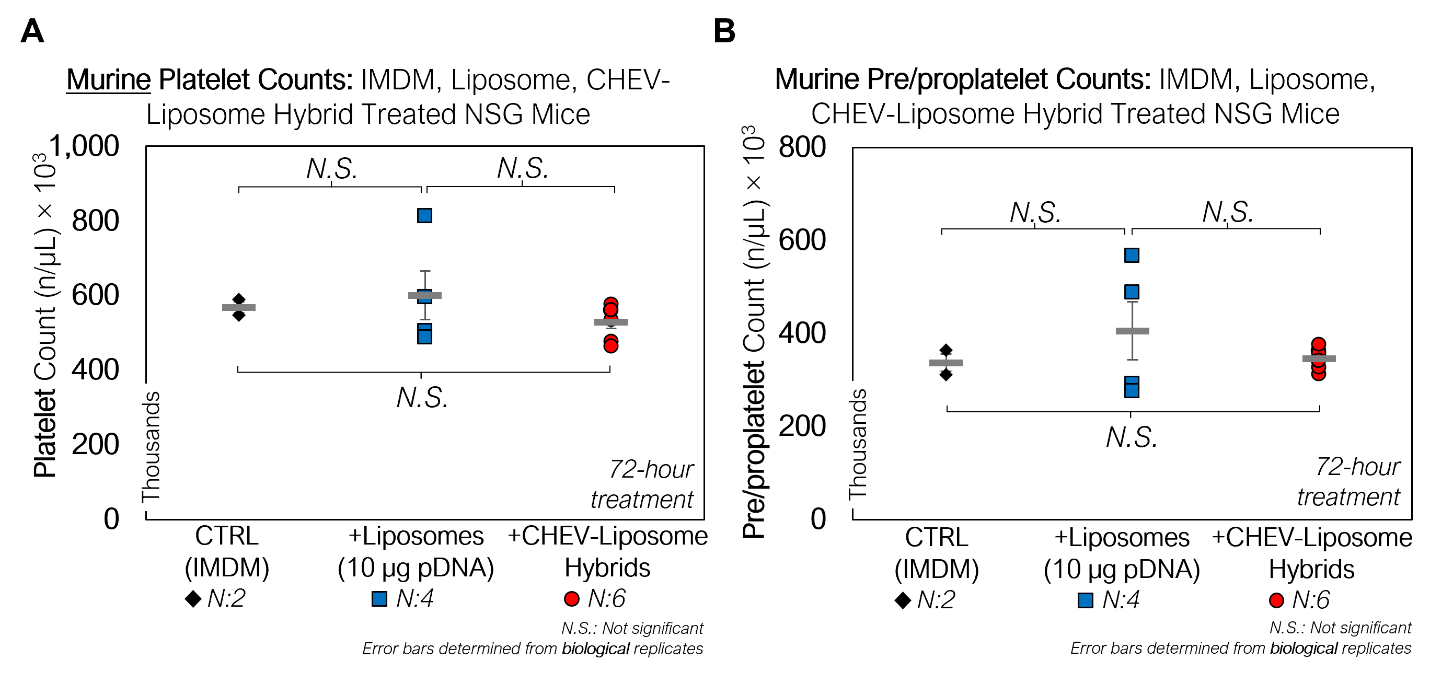


**Supplemental Figure S3. Impact of liposome, CHEV-liposome hybrid treatment on platelet counts.** A) Platelet and B) pre/proplatelet counts in NSG peripheral blood 72-hours following treatment with IMDM (media control), pDNA-loaded liposomes, or CHRF EV-liposome hybrids. *N.S.: not significant. Analysis of Variance (ANOVA) was used for statistical comparisons for all animal experiments.*

##
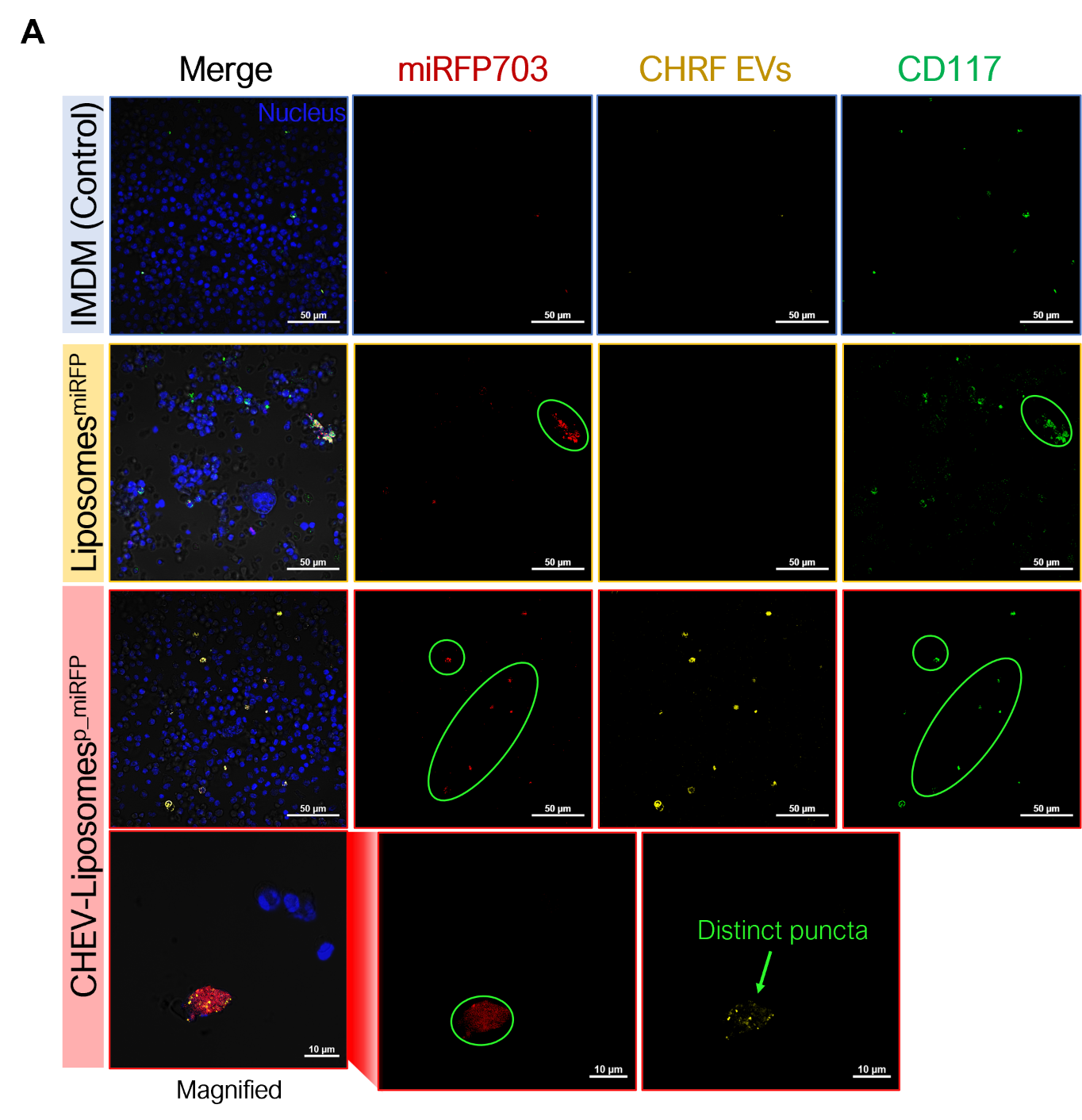
Supplementary Figure S4

**Supplemental Figure S4. Flushed marrow (femur) cells from mice treated with pDNA –loaded CHEV-liposome hybrids exhibit expression of miRFP703 from delivered plasmid.** Flushed RBC-depleted marrow cells from mice treated with either pDNA-loaded liposomes (pLifeAct-miRFP703) or pDNA-loaded CHEV-liposome hybrids and a media (IMDM)-treated control were immunostained for CD117+ HSPCs (green) and screened for miRFP703 expression (red), and CHEVs (yellow). Colocalization of murine HSPCs and miRFP703 indicated with light green circles and nuclei are shown in blue.

## Supplementary Figure S5

**
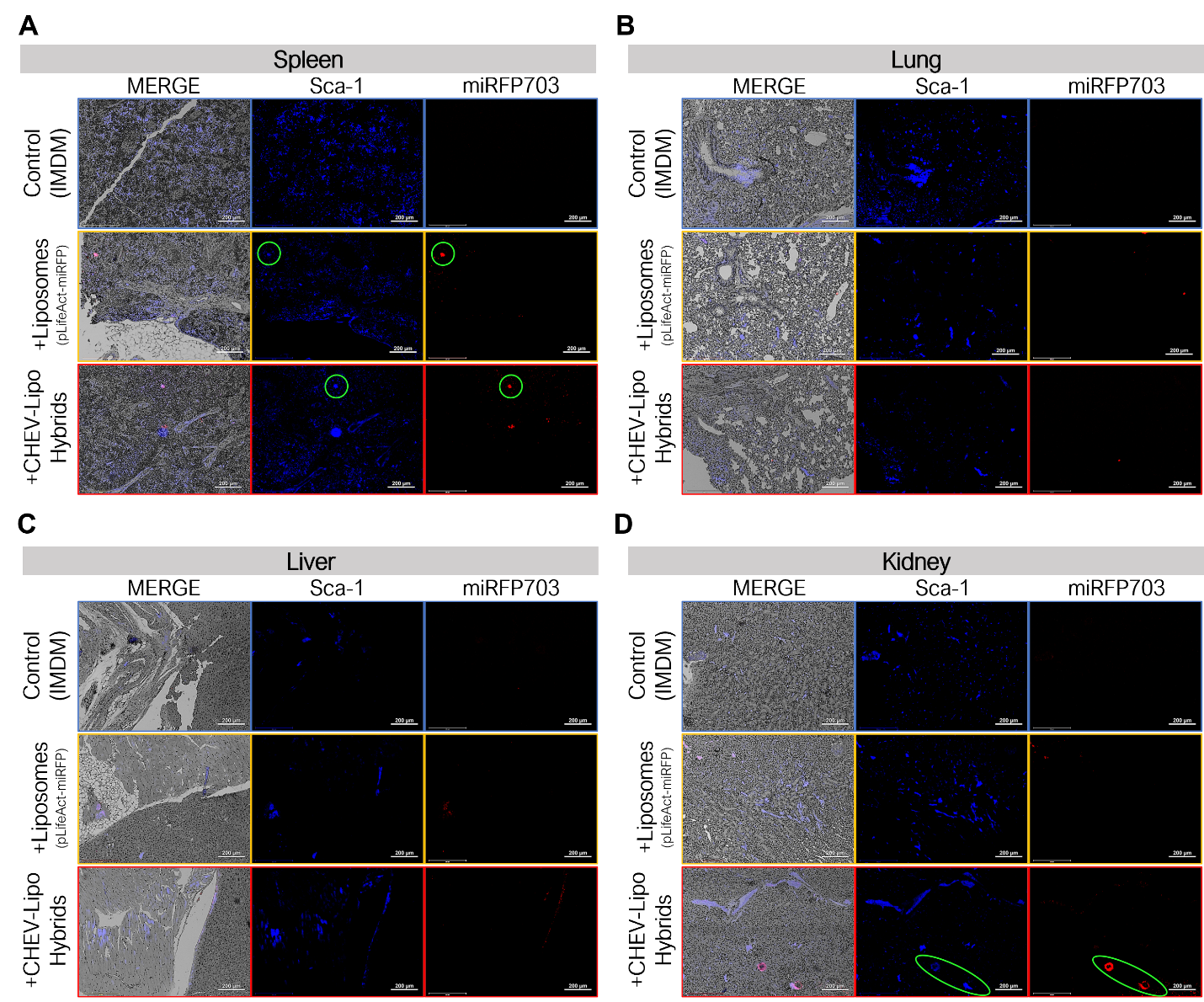
**

**Supplemental Figure S5. Histological analysis of various Sca-1 immunostained tissues excised from pDNA-loaded liposome and CHEV-liposome hybrid-treated mice.** Lungs, spleen, kidneys, and liver from 72-hour-treated mice were fixed (10% neutral-buffered formalin), sectioned, immunostained and assessed for structure (gray- DIC) and presence of Sca-1+ cells (blue), miRFP703 expression (red) and PKH26 arising from administered CHEV hybrids. Colocalization of murine HSPCs and miRFP703 indicated with light green circles. Scale bars: 200-µm.

## Supplementary Figure S6

**
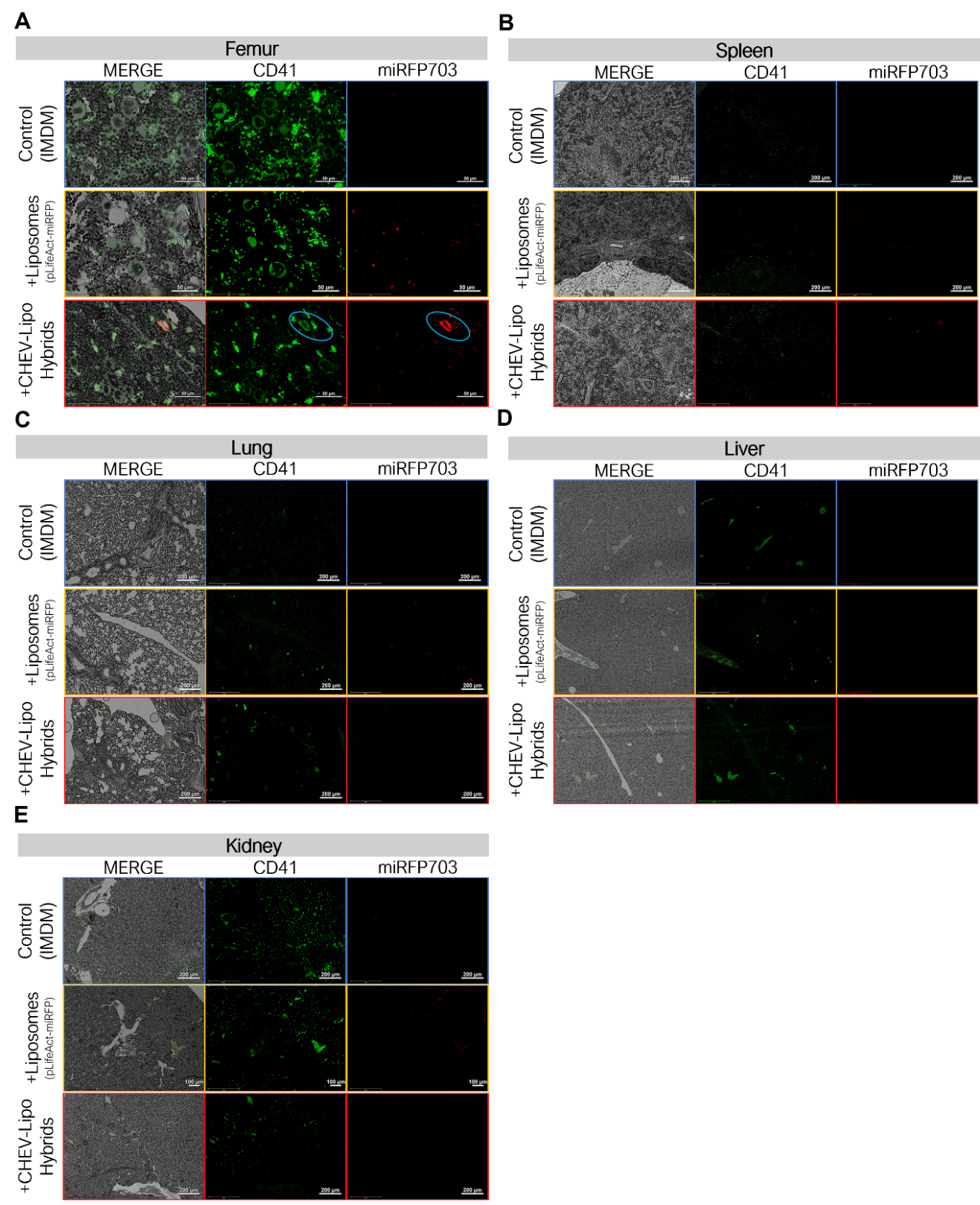
**

**Supplemental Figure S6. Histological analysis of various CD41 immunostained tissues excised from pDNA-loaded liposome and CHEV-liposome hybrid-treated mice.** Lungs, spleen, kidneys, and liver from 72-hour-treated mice were fixed (10% neutral-buffered formalin), sectioned, immunostained and assessed for structure (gray- DIC) and presence of CD41+ cells (green), miRFP703 expression (red) and PKH26 arising from administered CHEV hybrids. Colocalization of murine CD41+ megakaryocytes and miRFP703 indicated with light blue circles. Scale bars: 200-µm.
